# EEG Resting State Functional Connectivity Analysis in Children with Benign Epilepsy with Centrotemporal Spikes

**DOI:** 10.3389/fnins.2016.00143

**Published:** 2016-03-31

**Authors:** Azeez Adebimpe, Ardalan Aarabi, Emilie Bourel-Ponchel, Mahdi Mahmoudzadeh, Fabrice Wallois

**Affiliations:** ^1^INSERM U 1105, CURS, Centre Hospitalier Universitaire Amiens-PicardieAmiens, France; ^2^INSERM U 1105, EFSN Pédiatriques, Centre Hospitalier Universitaire Amiens-PicardieAmiens, France

**Keywords:** children epilepsy, centrotemporal spikes, resting sate, functional connectivity, phase synchronization, graph theory

## Abstract

In this study, we investigated changes in functional connectivity (FC) of the brain networks in patients with benign epilepsy with centrotemporal spikes (BECTS) compared to healthy controls using high-density EEG data collected under eyes-closed resting state condition. EEG source reconstruction was performed with exact Low Resolution Electromagnetic Tomography (eLORETA). We investigated FC between 84 Brodmann areas using lagged phase synchronization (LPS) in four frequency bands (δ, θ, α, and β). We further computed the network degree, clustering coefficient and efficiency. Compared to controls, patients displayed higher θ and α and lower β LPS values. In these frequency bands, patients were also characterized by less well ordered brain networks exhibiting higher global degrees and efficiencies and lower clustering coefficients. In the β band, patients exhibited reduced functional segregation and integration due to loss of both local and long-distance functional connections. These findings suggest that benign epileptic brain networks might be functionally disrupted due to their altered functional organization especially in the α and β frequency bands.

## Introduction

Benign epilepsy with centrotemporal spikes (BECTS) is the most common idiopathic epileptic syndrome with a prevalence of 8–20% of pediatric patients with epilepsy (Holmes, 1993; Wirrell, 1998; Panayiotopoulos, 1999). In BECTS, although interictal spikes arise primarily within centrotemporal regions, there is growing evidence that abnormal functional networks in BECTS patients, like other types of focal epilepsy, are not restricted to the epileptogenic region as revealed by the functional connectivity (FC) analysis of the brain networks (Kramer and Cash, 2012; Laufs, 2012; Adebimpe et al., 2015b). Moreover, our previous studies have shown that functional organization of the brain networks in BECTS patients largely differs from normal brain in presence or absence of interictal epileptic discharges (IES) (Adebimpe et al., 2015b, 2016).

Over the past decade, graph-theoretical analysis of resting state FC in EEG and MEG data have gained attention in healthy subjects (Deco and Kringelbach, 2014), and in patients suffering from various diseases (Prinz, 2008; Stam et al., 2009; Zhang et al., 2014). In our previous study using graph metrics we found that the brain networks in BECTS patients show functionally disrupted connectivity patterns (Adebimpe et al., 2015b). However, the main shortcoming of that study was that the FC analysis and graph metrics were estimated in the sensor space that might not provide information on the overall functional organization of the cortical regions mainly because scalp EEG electrodes detect spatially averaged overlapping EEG signals from several brain sources. Moreover, our previous study focused on global clustering coefficient and path length.

In the present study, we investigated changes in brain FC including local and regional graph metrics in BECTS patients compared to healthy controls in various frequency bands under the eyes-closed resting condition. Abnormal patterns of resting-state EEG source FC in patients were determined by using lagged phase synchronization (LPS), a non-linear connectivity measure implemented in the eLORETA software (http://www.uzh.ch/keyinst/eLORETA/). Graph theory was also used to characterize FC by estimating network centrality, functional segregation and integration. We further investigated whether functional brain networks in BECTS patients displayed altered network efficiency and disrupted local neural processing (segregation and integration) in comparison to healthy controls.

## Materials and methods

### Subjects

This study was performed on 11 young patients (9.65 ± 2.36 years) with benign childhood epilepsy with right centrotemporal spikes (see Table 1 and Adebimpe et al., 2015a, for more information). None of the patients presented any other neurological disorders at the time of the study, which was conducted at Amiens University Hospital (Amiens, France) and approved by the hospital's ethics committee (CPP Nord-Ouest 2, approval No. 2011-A00782-39). We also recruited 12 healthy subjects in the same age range (9.27 ± 1.70 years) as controls. Written consent approved by the ethics committee was obtained from parents/caregivers.

**Table 1:** **Characteristics of the control and patient groups**.

**Control group**			**Patient group**					
**Subject**	**Age (years)**	**EEG duration (min)**	**Patient**	**Age (years)**	**EEG duration (min)**	**Neuropsychological assessment**	**Description of ictal EEG**	**Medication**
1	6.73	16	1	12.63	50	Normal	Partial seizure	Sodium valproate
2	11.28	19	2	12.64	17	Normal	Partial seizure	Sodium valproate
3	10.48	19	3	9.25	44	Attention deficit	Generalized tonic-clonic seizure	Oxcarbezepine
4	10.66	17	4	6.03	43	–	Brachiofacial nocturnal seizure	Oxcarbezepine
5	7.39	13	5	10.47	50	Attention deficit	Partial seizure	Sodium valproate
6	7.31	20	6	7.16	14	–	Brachiofacial nocturnal seizure	Sodium valproate
7	11.92	30	7	8.51	30	Attention deficit	Nocturnal seizure	–
8	8.44	75	8	13.16	20	Normal	Generalized tonic-clonic seizure	Sodium valproate
9	9.36	28	9	9.67	15	Language deficit	Generalized tonic-clonic seizure	Lamotrigine
10	9.48	45	10	7.79	23	Normal	Generalized tonic-clonic seizure	Micropakine
11	10.32	18	11	8.91	16	Normal	Generalized tonic-clonic seizure	Trileptal
12	7.98	20		-	–	-	–	–
Mean ± SD	9.3 ± 1.7	27 ± 17		9.6 ± 2.4	29.7 ± 14			

### EEG recording and pre-processing

EEG data were recorded with a high density recording system (ANT, Netherlands) based on the international 10–10 system at a sampling rate of 256 Hz under the eyes-closed resting condition. EEG data were first digitally re-referenced to an average reference, z-scored, and band-pass filtered between 0.5 and 40 Hz to exclude high-frequency noise including muscle activities. EEG portions with occular and movement artifacts were identified automatically using a thresholding method (threshold was set to the mean of the z-score distribution for each channel) as implemented in Fieldtrip software (Oostenveld et al., 2010; tutorial:visual_artifact_rejection – FieldTrip^1^) and rejected by visual inspection. No ECG artifacts were visually observed in any of the EEG recordings. The artifact-free portions of the EEG data were partitioned into 2-s quasi-stationary segments required for spectral analysis with a frequency resolution of 0.5 Hz. Five EEG segments of 2 s were randomly selected for each patient (PAT) and healthy control (CON). The EEG segments selected for patients included no centrotemporal spikes.

### EEG source connectivity analysis

We first used the exact Low Resolution Electromagnetic Tomography (eLORETA) method (Pascual-Marqui, 2007a) to identify the average location of interictal spike sources in patients. EEG source connectivity analysis was then performed using eLORETA by restricting the source space within the gray matter including 6239 voxels with a 5-mm spatial resolution. The Montreal Neurologic Institute average MRI brain (MNI152) (Fonov et al., 2011) with anatomical labels corresponding to Brodmann areas was used as the realistic head model to compute the lead field. The 84 commonly used Brodmann areas were chosen as regions of interests (ROIs) for connectivity analysis between the centroids of the ROIs.

To analyze the FC we computed LPS (Pascual-Marqui, 2007b) between ROIs. This measure has been shown to be less sensitive than other techniques to non-physiological signals including artifacts and the volume conduction effect (Pascual-Marqui et al., 2011). For each subject, four FC matrices were computed in four frequency bands, δ (0.5–3.5 Hz), θ (4–8 Hz), α (8.5–13 Hz), and β (14–30 Hz). For each subject and each frequency band, an average FC matrix was obtained over the five EEG segments selected for the subject and was used to compute graph metrics.

### Graph theoretical analysis

From each FC matrix, we extracted three graph measures to investigate functional integration and segregation between brain networks in patients compared to controls (for mathematical definitions see (Rubinov and Sporns, 2010): network degree (K), a measure of node centrality, global efficiency (E) and clustering coefficient (C), measures of functional integration and segregation in large-scale brain networks, respectively.

The graph measures were calculated using the brain connectivity toolbox (Rubinov and Sporns, 2010). To make connectivity matrices comparable across subjects, an individual optimal threshold was needed to convert each FC matrix to a binary adjacency matrix. This step was necessary to ensure that all graphs had equal connection densities within the small world network range (Bassett et al., 2006). Moreover, the optimal thresholds have been shown to reduce the number of false-positive edges and minimize the noise (Drakesmith et al., 2015). To obtain an optimal threshold for each subject and frequency band, we first set the threshold to one standard deviation above the median connectivity value. The threshold was iteratively adjusted to satisfy two conditions: (i) the mean network degree had to be less than 2log(N), where N was the total number of nodes, and (ii) at least 95% of nodes must be connected to one or more nodes (Bassett et al., 2006; Erdős and Rényi, 2013). Using the optimal thresholds, the FC matrices were thresholded and binarized to calculate the network degree (K), clustering coefficient (C), and global efficiency (E). The global value for each graph metric was calculated by the average over the whole nodes for a wide range of thresholds from 0.2 to 0.7 with 0.05 increments to investigate differences between the two groups.

### Statistical analysis

Group differences in FC and global network metrics were statistically evaluated. Statistical comparisons between patients and controls were performed using non-parametric permutation *t*-tests with *p* < 0.05 (Bonferroni corrected for multiple comparisons). We further used *Post-hoc t-*tests to explore the directionality of effects over conditions with *p* < 0.05. A total of 1000 permutations were used to determine the significance level for each test (Maris and Oostenveld, 2007). The results were then projected onto a 3D surface using BrainNet (Xia et al., 2013).

## Results

### EEG source functional connectivity

**Figure 1:**
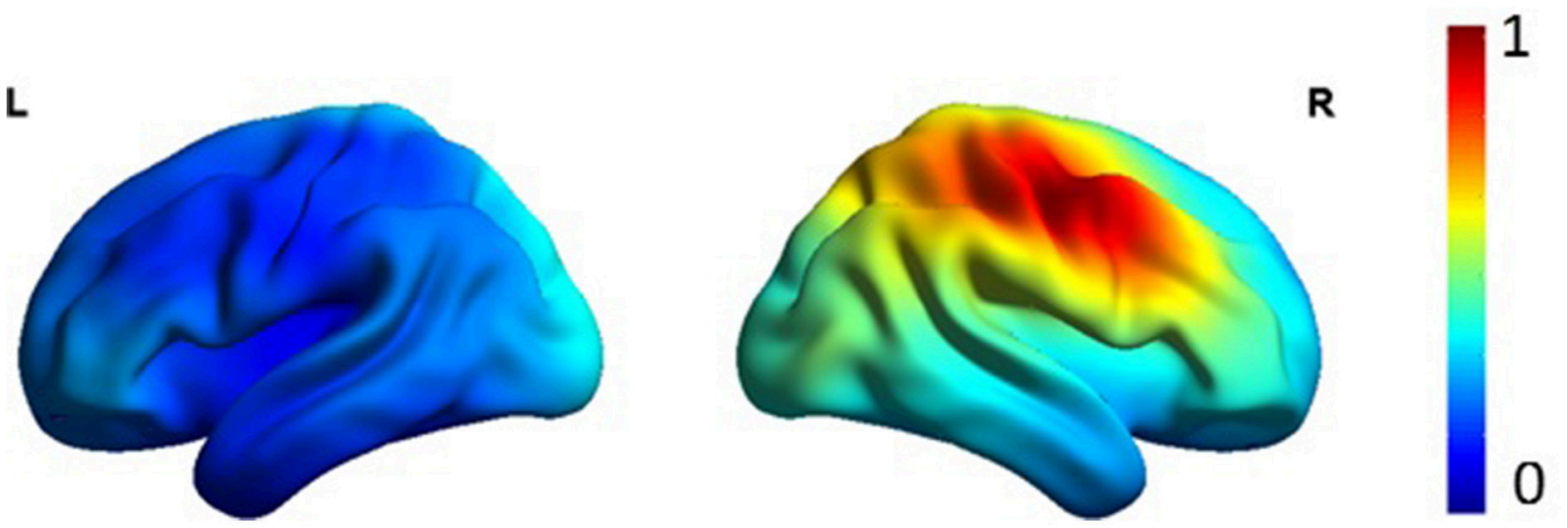
**Average eLORETA source localization of interictal spikes in patients**. The squared magnitude of the current density is color coded from dark blue (zero) to dark red (one).

**Figure 2:**
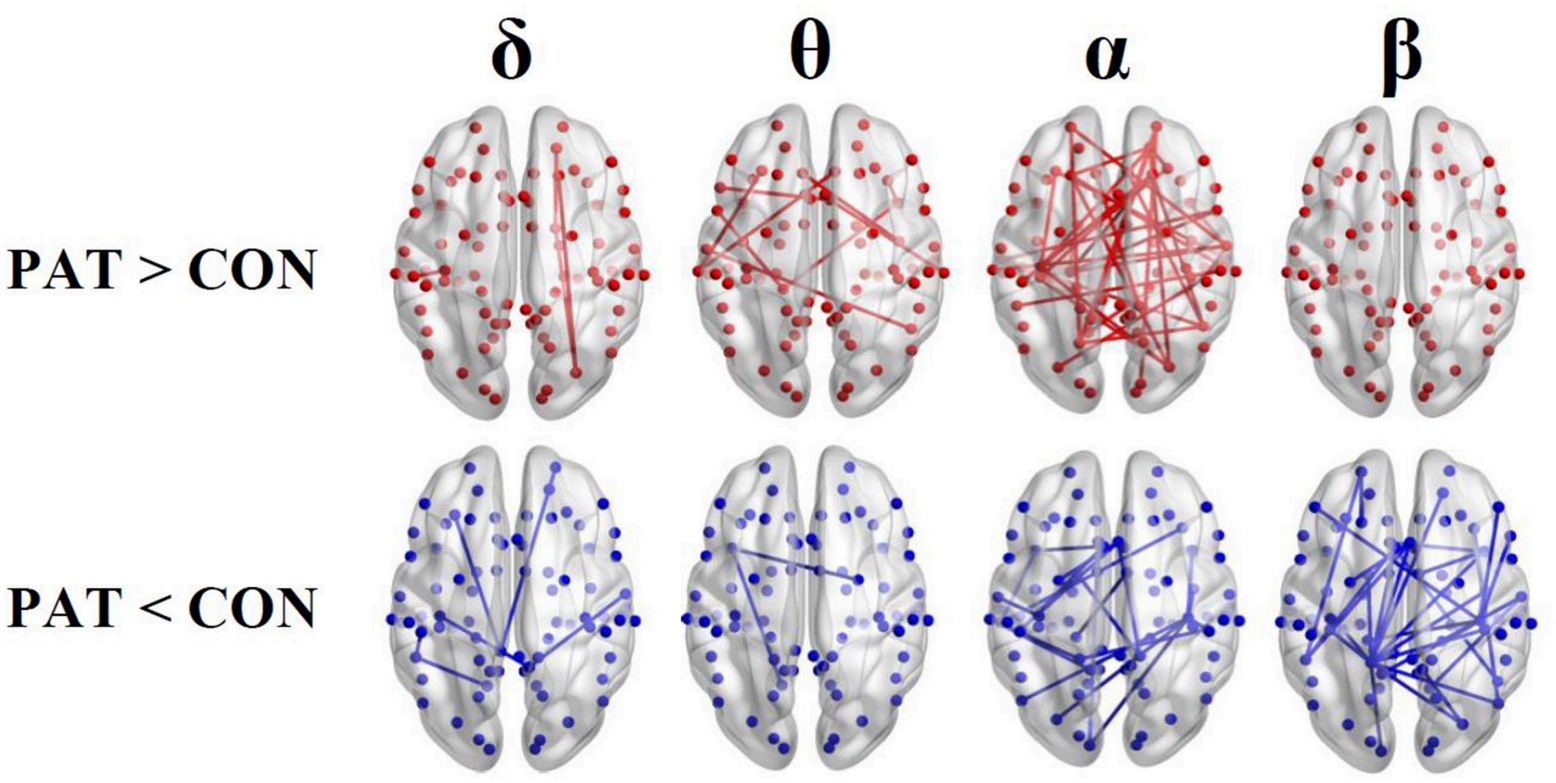
**Results of the functional resting-state source connectivity analysis with the seeds located at the Brodmann area centroids**. The upper and lower rows indicate significantly higher (red lines) and lower (blue lines) connectivity values in patients (PAT) compared to controls (CON), respectively.

Figure 1 shows the average localization of interictal spikes in patients. As shown, all the patients exhibited an epileptic focus at the right hemisphere with a spatial extent restricted to the right central areas. Results of lagged connectivity differences between patients and healthy controls in all frequency bands are summarized in Figure 2. The major differences between the two groups were observed in the α and β bands. Compared to healthy controls, patients showed significantly increased α LPS over most cortical regions. There was also significantly reduced α LPS in the temporal and right centrotemporal areas. In contrast with the α band, significantly lower LPS values were observed in the β band in almost all brain regions of patients. In patients compared to controls, δ LPS values were significantly higher between the right anterior and posterior areas along the midline and lower between the temporal and posterocentral areas. In the θ band, patients displayed higher LPS values between temporal and central areas, mostly over the left hemisphere.

### Global network measures

**Figure 3:**
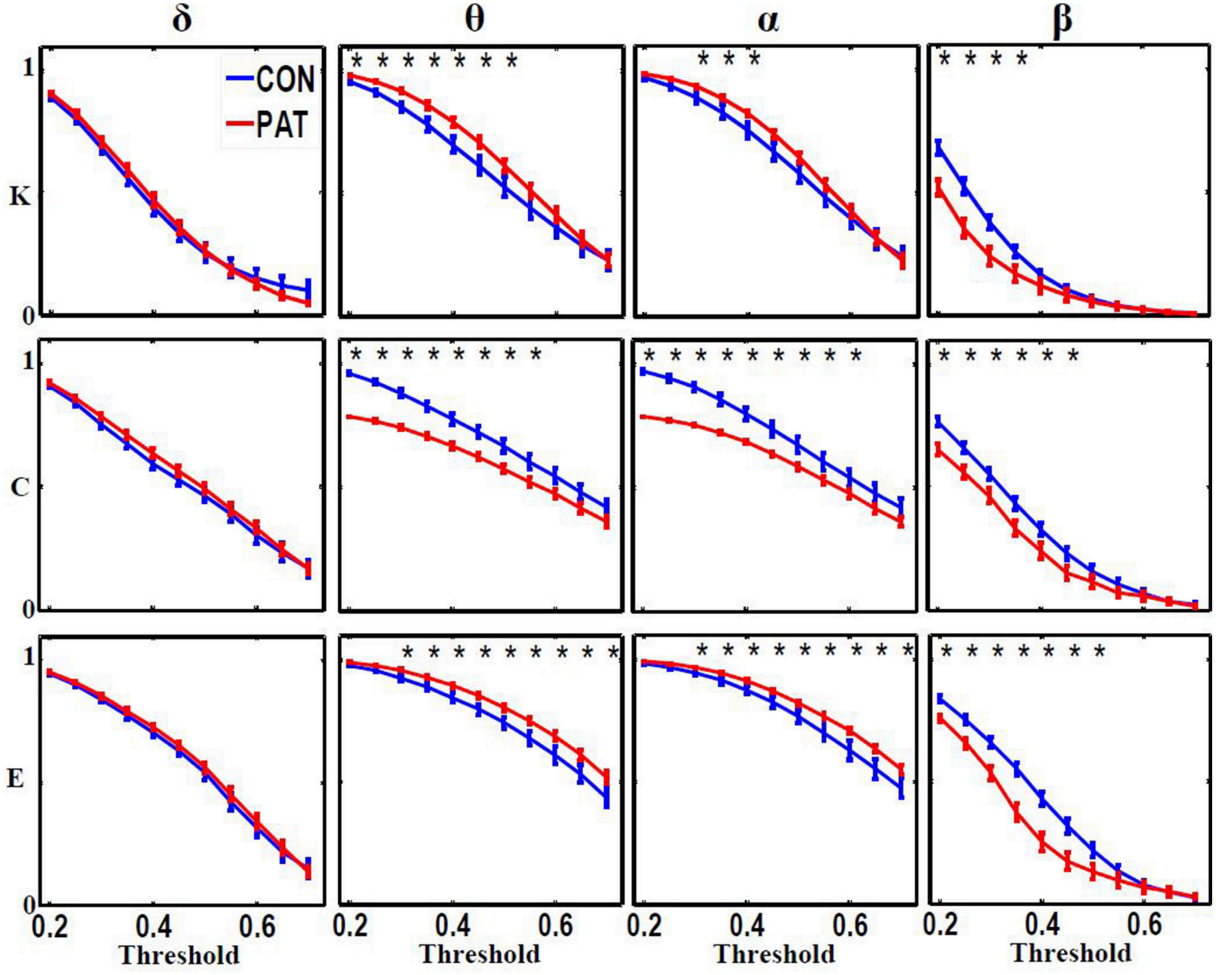
**Global network degree (K), clustering coefficient (C) and efficiency (L) as a function of threshold values for each frequency band**. The error bars represent the standard error of the mean with 95% confidence intervals and ^*^ indicate significant differences between PAT and CON.

Figure 3 shows the global metric values over thresholds between 0.2 and 0.7. Patients presented higher global network degree and efficiency and lower clustering coefficients in the θ and α bands. At higher frequencies (β), patients were characterized by lower values for all three measures over thresholds up to 0.4. At very low frequency (δ), no significant differences were observed over all of the threshold range between patients and healthy controls. Table 2 presents the optimal thresholds used to compute the global network degree (K), global clustering coefficient (C), and global efficiency (E) for both groups.

**Table 2:** **Mean values (with range at 95% confidence interval) of threshold (T), degree (K), clustering coefficient (C), and global efficiency (E) computed for each group and frequency band**.

	**Frequency band**	**T**	**K**	**C**	**E**
Controls (CON)	δ	0.54 ± 0.05	0.16 ± 0.05	0.42 ± 0.03	0.48 ± 0.04
	θ	0.70 ± 0.05	0.14 ± 0.05	0.44 ± 0.04	0.47 ± 0.05
	α	0.71 ± 0.05	0.15 ± 0.05	0.45 ± 0.03	0.46 ± 0.04
	β	0.38 ± 0.02	0.18 ± 0.01	0.46 ± 0.00	0.53 ± 0.01
Patients (PAT)	δ	0.54 ± 0.02	0.17 ± 0.00	0.43 ±0.01	0.51 ± 0.01
	θ	0.72 ± 0.03	0.16 ± 0.01	0.43 ± 0.01	0.50 ± 0.01
	α	0.73 ± 0.02	0.15 ± 0.01	0.41 ± 0.02	0.49 ± 0.00
	β	0.32 ± 0.03	0.17 ± 0.00	0.42 ± 0.02	0.44 ± 0.01

### Nodal network degree and clustering coefficient

**Figure 4:**
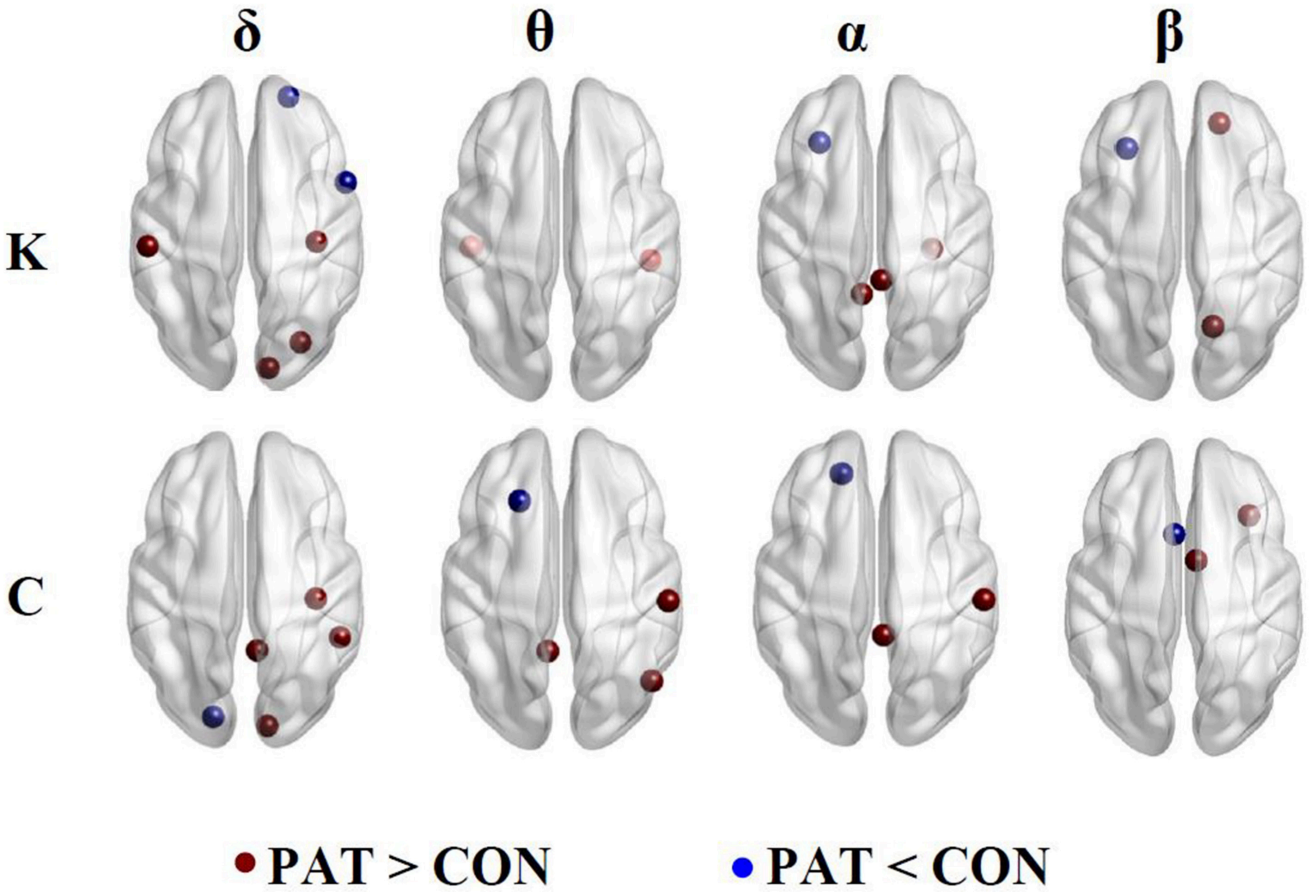
**Nodal network degree (K) and clustering coefficient (C)**. Brown and blue dots indicate higher and lower K and C in patients compared to controls, respectively.

Figure 4 illustrates the regions presenting statistically significant differences between patients and healthy controls for nodal network degree (K) and clustering coefficient (C) computed using optimal thresholds.

### Network degree (K)

In the δ band, patients, compared to controls displayed higher network degree values at the right somatosensory cortex, left motor cortex and right occipital lobe and decreased K at the right anterior frontal cortex and opercula frontal regions. In the θ band, patients showed significantly higher K values at the right inferior temporal and left primary auditory cortex. In the α band, patients displayed a higher K at the right posterior cingulate cortex, right temporal and left isthmus of the cingulate cortex. In both the α and β bands, patients displayed lower K values at the left frontal cortex. In the β band, patients displayed higher K values at the right superior parietal and left prefrontal cortex.

### Clustering coefficient (C)

In the δ band, patients displayed higher clustering coefficient (C) at the right primary motor and visual cortex, right cingulate gyrus, and lower C values at the left primary auditory cortex. In the θ band, patients displayed higher C values at the right postcentral, right angular gyrus and left dorsal cingulate gyrus and lower C in the left intermediate frontal region. In the α band, patients displayed higher C values at the right primary motor and right posterior cingulate cortices, and, in the β band, displayed a decreased C at the right orbital frontal with higher C values in the frontal regions.

## Discussion

In this study, we investigated abnormal patterns of resting-state EEG FC in BECTS patients using graph metrics in various frequency bands. Our results suggest that the functionality of brain networks in BECTS patients is altered, particularly in the α and β bands. Compared to healthy subjects, BECTS patients were characterized by widespread higher and lower phase synchronization values in the α and β bands, respectively.

In the θ and α bands, significantly lower global clustering coefficient and higher network degree and efficiency were observed in patients. In contrast, compared to controls, patients displayed significant lower global metrics in the β band. In the δ band, no significant differences in global graph metrics were observed between patients and controls.

### Global functional integration and segregation

The human brain is very complex, comprising inhibitory and excitatory circuits that interact by integrating information at local and global levels. The functional segregation and integration of brain networks is expected to be balanced in healthy subjects. It has been shown that the normal brain has a small world functional topology, which can efficiently combine functionally specialized (segregated) modules with intermodular (integrating) links (Bassett and Bullmore, 2006). This type of organization reflects an optimal balance between functional integration and integration (Bassett and Bullmore, 2006; Ponten et al., 2007).

In BECTS patients, however, we found that the functional organization of brain networks was altered in a frequency dependent manner. Our results indicate that the interictal state in BECTS patients is less well ordered, displaying lower segregation (lower global clustering coefficient) and higher integration (higher global efficiency and network degree) in the θ and α bands. This finding is in line with the results reported in our previous study (Adebimpe et al., 2015b) and other related studies (Clemens, 2004; Boor et al., 2007; Quraan et al., 2013). As a characteristic feature of BECTS, the alteration in brain functional organization might be explained by the abnormal significant increase in the power of θ oscillations (Clemens, 2004; Clemens et al., 2010; Douw et al., 2010; Adebimpe et al., 2014, 2015a).

In the α band, the lower clustering coefficient and the higher network degree might be due to the loss of local connectivity between neighbor nodes in comparison to increased long distance connections in BECTS patients, as shown in Figure 2. The less ordered network configuration in patients with BECTS has also been reported in previous studies on epilepsy (Ponten et al., 2007; Quraan et al., 2013; Adebimpe et al., 2015b) and other neurological diseases (Liu et al., 2008; Stam et al., 2009; Wang et al., 2009; Zhang et al., 2011) compared to healthy controls. Our findings are consistent with a recent fMRI study (Song et al., 2015) reporting low local efficiency (an alternative to the clustering coefficient) and high global efficiency in BECTS patients. Our findings are also in agreement with those of Quraan et al. (2013), who reported low C and short L (high E) in patients with temporal lobe epilepsy (TLE) in the α band. In patients with focal epilepsy, however, higher clustering coefficients and longer path lengths have been reported in comparison to healthy subjects (Bernasconi et al., 2003; van Diessen et al., 2014; Taylor et al., 2015). Both TLE and focal epilepsy are known to be associated with abnormal structural brain alterations. In BECTS, there is no evidence of alterations in the brain structure.

In the β band, we observed a significant pruning of long- and short-distance functional connections in BECTS patients, who exhibited reduced clustering coefficient (representing functional segregation) and lower global efficiency and network degree (representing functional integration) compared to healthy controls. The frequency-dependent alterations in the brain functional organization in BECTS patients may constitute specific biomarkers of the benign epilepsy.

In healthy subjects, studies of large-scale brain FC have also shown that interactions between spatially distinct brain regions are frequency dependent. Delta, theta and alpha FC have been shown to be related to attention, learning, memory, and emotion processing (Knyazev, 2007; Bekkedal et al., 2011; Calmels et al., 2012). Alpha FC is also shown to be associated to motor performance in adults (Sauseng et al., 2005; Klimesch et al., 2007; Palva and Palva, 2007). Based on these findings, we postulate that in BECTS patients alterations in brain FC in different frequency bands may cause cognitive, mental, memory and attention impairment (Baglietto et al., 2001; Datta et al., 2013; Kim et al., 2014; Verrotti et al., 2014).

### Local changes in functional connectivity

Our FC analysis at Brodmann areas revealed higher network degree and clustering coefficient in epileptogenic areas, including centrotemporal, premotor, and somatosensory regions in the θ and α bands in BECTS patients. We also found higher degree in the posterior cingulate cortex in patients in the θ and α bands. This finding is consistent with the results of previous studies (Boor et al., 2007; Besseling et al., 2013; Tang et al., 2014) indicating higher activity in the supplementary motor region in epileptic patients. Our results also confirmed the higher phase synchronization values in the regions with increased local segregation neural processing (high clustering coefficient), especially in the primary motor, postcentral and posterior cingulate regions. Significantly, higher phase synchronization values were also observed in the central regions, including somatosensory cortex and motor cortex (Avanzini et al., 2012). However, a decreased network degree was observed in the right frontal cortex in the α and β bands, and a low clustering coefficient was also observed in the intermediate frontal region in the θ band. The frontal lobe is known to play a major role in the processing and execution of higher cognitive skills and behaviors (Stuss, 2011) and children with benign epilepsy have been found to present cognitive deficits and impaired mental activity (Ay et al., 2009; Datta et al., 2013; Verrotti et al., 2014). Furthermore, the right auditory network, including right temporal, parietal and left auditory cortex (in θ band), also showed altered (higher) functional integration (network degree) in patients, which could affect auditory processing in both hemispheres, resulting in language processing deficits in BECTS patients (Naganuma et al., 1994; Tomé et al., 2014; Filippini et al., 2015).

Several fMRI studies have reported impairments in different brain networks in BECTS patients (Kim et al., 2014; Yang et al., 2015; Xiao et al., 2015a,b). We also found differences in brain functional organization between BECTS patients and healthy controls. Although some studies reported associations between EEG rhythms and fMRI maps but there are some inconsistencies (Laufs et al., 2003; Mantini et al., 2007; Bridwell et al., 2013) due to underlying differences between these modalities as EEG measures direct neuronal activity while fMRI records indirectly brain activity.

In our previous work (Adebimpe et al., 2015b), we compared patients and controls using three graph metrics, degree, clustering coefficient and path length computed using sensor-level EEG data. In that study, we used the Phase Locking Value (PLV) as a measure of FC between electrodes to explore the global topology and dynamics of functional interactions between large-scale brain regions during the resting state over a range of frequencies. The results obtained using the sensor- and source-level connectivity analyses were consistent. In the present study, we also found higher clustering coefficients and shorter path lengths in the theta and alpha bands in BECTS patients compared to controls. However, the FC analysis in the sensor space is more sensitive to artifacts of volume conduction especially using zero-lag connectivity measures like PLV. Moreover, statistical comparisons at the group level are less reliable at the sensor space because of variability in EEG electrode positions across subjects. Instead, FC estimated at the source level reflects actual interactions between brain areas.

### Methodological consideration and limitations

In this study, we used eLORETA, an improved version of LORETA, for source imaging. It has been reported that this technique has no localization bias even in the presence of structured noise (Pascual-Marqui, 2007a). However, this technique like other EEG localization method is vulnerable to artifacts of volume conduction, head-modeling errors and EEG noise (Grech et al., 2008). Moreover, since the results of LORETA are model dependent, it may not accurately represent the neuronal origins of the brain activity (Hata et al., 2016).

Another major challenge is the choice of source FC analysis. The majority of zero-lag connectivity measures such as correlation, coherence, and PLV are developed based on scalp sensors and thus sensitive to volume conduction effect (Gross et al., 2001). In a MEG study, Ghuman et al. (2011) have shown that artifacts of volume conduction can result in increased false-positive PLVs. To reduce the effect of volume conduction on the results of the source connectivity analysis, we used the LPS to investigate FC in the source space (Pascual-Marqui, 2007b). This method has been successfully used to explore EEG source FC in epilepsy studies (Canuet et al., 2011; Vecchio et al., 2015; Hata et al., 2016). This connectivity measure is resistant to artifacts of volume conduction by excluding the instantaneous zero-lag contribution (Canuet et al., 2011; Vinck et al., 2011). However, like phase lag index, LPS's sensitivity to uncorrelated perturbation, which can turn phase lags into leads, has to be investigated especially when EEG noise is strong (Nolte et al., 2004; Stam et al., 2007; Vinck et al., 2011).

We further used a ROI-based approach, which is a common practice in neuroimaging studies to reduce variability in brain size and shape between individuals which might affect estimation of source FC (Schoffelen and Gross, 2009). This approach is based on the BA regions cytoarchitectonically defined in the Talairach atlas. The ROI for each of 84 BA regions was defined as a single centroid voxel (the closest to the center of each region). The ROI-approach is more efficient in reducing volume conduction artifacts than the voxel-by-voxel connectivity analysis as it has been shown that the spatial correlation between sources decays with increasing distance between them (Mehrkanoon et al., 2014).

Our results should be interpreted with caution because of the low sample size, limited number of EEG segments, and short EEG data lengths. Apart from technical limitations, collecting long EEG recordings from children under age 16 is difficult because they cannot adequately follow instructions. We further had to exclude artifactual EEG portions and segments with interictal spikes from our analysis. Therefore, for each subject only five segments were randomly selected for FC analysis. Further studies with larger samples and longer EEG recordings are required to confirm our findings.

## Conclusion

In this study, we investigated functional alterations in BECTS patients with BECTS. Compared to controls, patients were characterized by higher θ and α and lower β phase synchronization values. Our observations support previously reported evidence that alteration in BECTS brain networks is frequency-dependent, as patients showed lower clustering coefficients in the three frequency bands. Our findings suggest that benign focal epilepsy is associated with altered resting state FC that probably arises from disrupted topological organization of resting-state functional brain networks.

## Author contributions

Conceived and designed the experiments: AA, AA, FW. Performed the experiments: EB, MM. Analyzed the data: AA, AA. Contributed reagents/materials/analysis tools: AA, AA, EB, MM, FW. Wrote the paper: AA, AA, FW. Read and accepted the manuscript: AA, AA, EB, MM, FW.

### Conflict of interest statement

The authors declare that the research was conducted in the absence of any commercial or financial relationships that could be construed as a potential conflict of interest.

## Footnotes

^1^tutorial:visual_artifact_rejection – FieldTrip Available at: http://www.fieldtriptoolbox.org/tutorial/visual_artifact_rejection [Accessed May 15, 2015].
